# Supplementary material for: Gender differences in job satisfaction among disabled workers
Source: PLoS One. 2021 Jun 4;16(6):e0252270. doi: 10.1371/journal.pone.0252270 (PMC8177467; doi:10.1371/journal.pone.0252270)
Supplement: Attachment — Submitted filename: Reviewer report 03Aug20.pdf [file pone.0252270.s001.pdf]

Gender Differences in Job Satisfaction among Disabled Workers

Using a panel data from PSED, this paper examines the effects of gender on job satisfaction among disable workers in Korea. This paper addresses the gender differences in job satisfaction of disable workers which is an important and current issue in the literature of job/life satisfaction. It is a well-structured paper, with a steady focus – the gender differences are explored using different model specifications, including the control variables of work values and expectations (proxy). Most of my doubts and questions tend to be addressed quickly. This paper has a potential benefit to the readers of PLOS ONE. Nevertheless, this paper is not publishable in its present form due to the following major concerns:

1. The estimated coefficients of the probability model (random effect ordered probit model) are interpreted as the marginal effects. For example, "…the gender differentials in job satisfaction decrease from 0.225 (Model 3) to 0.222 (Model 4) …" (page 21, para.2, line 6-7). Then, the authors concluded that the work values able to reduce partially the gender gap in job satisfaction (see Abstract, line 5-8).

   This might be misleading as the magnitude of an estimated coefficient of a probability model are not interpretable directly. The marginal effect of a probability model is a function of more than the estimated coefficients (see Long, 1997). The estimated coefficients need to be supplemented by others such as predicted probabilities.

   Long, J.S. (1997). Regression models for categorical and limited dependent variables. SAGE Publications: Thousand Oaks, California.

2. What is the goodness of fit of the estimated model? Does the random effect relevant in the estimated models? It will be a great help if the authors could present the results of goodness of fit tests on the estimated models. For example, the general specification test, hit-miss table (percentage correctly predicted for each outcomes of job satisfaction), multicollinearity and others. We need to convince the readers that the estimated models do not suffer problems in the estimation.

Other minor concerns are:

1. Label the variable of JS_{it}^{*} in Equation (1).

2. The use of notation of j_h and j in Equation (3). What is the different between j_h and j? If there is difference, please spell out what is j.

3. It is stated that "…k is the set of cutpoints $k_1, k_2, \dots, k_4$;" (page 17, para.4, line 1-2). From Equation (3), since there are five observed outcomes of job satisfaction, we have five cutpoints (instead of four), i.e., $k_0, k_1, k_2, \dots, k_5$. For example, when j=1, k=0; when j=5, k=5, as shown by Equation (3). Please clarify.

4. The Equation could be clearer if it is expressed as:

$$\Pr(JS_{it} = j) = \Pr\left(k_{j-1} < x_{it}\beta + v_i + \epsilon_{it} \leq k_j\right)$$
$$= \phi\left(k_j - x_{it}\beta - v_i\right) - \phi(k_{j-1} - x_{it}\beta - v_i)$$

5. Table 1 could be clearer if in the second column, the first row is interchangeable with the second row, such as:

Variable	Males (N=4,543)		Females (N=1,528)	
	Mean	Standard deviation	Mean	Standard deviation
:	:	:	:	:
:	:	:	:	:

Please note that there is a typo error in Table 1: "Jab satisfaction".

6. The literature review is extensive; however, it could be further improved by including reviews on the recent studies (2017 and onwards) in particular, the studies on the proxy of expectations and work values.
