# Supplementary material for: Gender differences in job satisfaction among disabled workers
Source: PLoS One. 2021 Jun 4;16(6):e0252270. doi: 10.1371/journal.pone.0252270 (PMC8177467; doi:10.1371/journal.pone.0252270)
Supplement: Attachment — Submitted filename: Reviewer report 22Feb21.pdf [file pone.0252270.s003.pdf]

Gender Differences in Job Satisfaction among Disabled Workers

The revised paper has improved substantially and most of the concerns are addressed satisfactorily. This paper indeed has a potential benefit to the readers of PLOS ONE. There are only a few minor concerns which the authors might wish to address with:

- The variable, JS_{it} , need to be labelled as well (line 245). For instance, "…probability that JS_{it} (i.e., the reported job satisfaction) is equivalent …"
- The specification of cut points seems inaccurate. "… $\boldsymbol{\kappa}$ is a set of cut points $\boldsymbol{\kappa}_1, \boldsymbol{\kappa}_2, \dots, \boldsymbol{\kappa}_4$; …" (line 250). This specification does not match with the Equation (3) in terms of *j*. According to Long (1997), the specification should be "…$\boldsymbol{\kappa}$ is a set of cut points $\boldsymbol{\kappa}_0, \boldsymbol{\kappa}_2, \dots, \boldsymbol{\kappa}_5$ $(where\ \boldsymbol{\kappa}_0 = -\infty\ \&\ \boldsymbol{\kappa}_5 = +\infty)$; …". This will match the Equation (3).
- It is stated that "…Our findings confirm that men are as happy in their workplace as women on average …" (line 324-325). From Table 3, it shows that women are happier than men. Please check.
- The use of the words, "…statistically insignificantly…" (line 340) seems weird. Please rewrite this sentence.
- The LR test statistics in Table 3 (Model 1) seem to incorrectly report. The extremely high values of LR test statistics (1388.089) appears to contradict with the low Wald chi-squared test statistics (2.325). Both LR test and Wald test are overall fit tests. They normally would not have huge differences in their test statistics. Please check.
- Title of Table 3, 4 and 5 include the word, "standard error" in parenthesis. How about delete it and include a footnote under the tables, such as "The values in parenthesis represent standard error"?
