# Supplementary material for: Gender differences in job satisfaction among disabled workers
Source: PLoS One. 2021 Jun 4;16(6):e0252270. doi: 10.1371/journal.pone.0252270 (PMC8177467; doi:10.1371/journal.pone.0252270)
Supplement: Attachment — Submitted filename: Response to Reviewers.docx [file pone.0252270.s002.docx]

**Reviewer #1:**

Using a panel data from PSED, this paper examines the effects of gender on job satisfaction among disable workers in Korea. This paper addresses the gender differences in job satisfaction of disable workers which is an important and current issue in the literature of job/life satisfaction. It is a well-structured paper, with a steady focus – the gender differences are explored using different model specifications, including the control variables of work values and expectations (proxy). Most of my doubts and questions tend to be addressed quickly. This paper has a potential benefit to the readers of PLOS ONE. Nevertheless, this paper is not publishable in its present form due to the some major concerns (pls refer to the uploaded reviewer report for details).

**Comment 1-1:** The estimated coefficients of the probability model (random effect ordered probit model) are interpreted as the marginal effects. For example, “…the gender differentials in job satisfaction decrease from 0.225 (Model 3) to 0.222 (Model 4) …” (page 21, para.2, line 6-7). Then, the authors concluded that the work values able to reduce partially the gender gap in job satisfaction (see Abstract, line 5-8). This might be misleading as the magnitude of an estimated coefficient of a probability model are not interpretable directly. The marginal effect of a probability model is a function of more than the estimated coefficients (see Long, 1997). The estimated coefficients need to be supplemented by others such as predicted probabilities. Long, J.S. (1997). Regression models for categorical and limited dependent variables. SAGE Publications: Thousand Oaks, California.

***Response:*** As stated by the reviewer, a separate table reporting marginal effects (Table 4) has been organized and related explanations have been reinforced. In relation to comparing the coefficient estimates of the gender variables across different models, we attempted to discuss the comparison based on marginal effects at ***page 15*** (Section 4). Thank you very much for this comment.

**Comment 1-2:** What is the goodness of fit of the estimated model? Does the random effect relevant in the estimated models? It will be a great help if the authors could present the results of goodness of fit tests on the estimated models. For example, the general specification test, hit-miss table (percentage correctly predicted for each outcomes of job satisfaction), multicollinearity and others. We need to convince the readers that the estimated models do not suffer problems in the estimation.

***Response:*** As pointed out by the reviewer, additional tests, related to the models, were conducted and test results were included at the end of Table 3. 1) By testing the null hypothesis of **H0: signa^2=0** through the LR test, we confirmed that random-effects model is more desirable than pooled one. 2) The overall significance of the models estimated was checked through the Wald test, confirming that unrestricted models in all of the three different specifications are valid. 3) Finally, we measured the hit-miss ratio to see how well the estimated model predicts the actual dependent variable. From these results, it seems appropriate to apply the random effects model, and the models estimated in this study are considered to be generally significant.

In addition, we are grateful for the minor suggestions, and I would like to say that we have made appropriate modifications to the parts the referee has mentioned. In particular, the recent research related to this study, **Perugini and Vladisavljevic (2019)**, has been included at the end of the section 2.1. And as appeared in Equation (2), the number of cut points to be estimated is “the observed outcomes of job satisfaction – 1”.

**Reviewer #2:**

The paper examines gender differences in job satisfaction among disabled workers using random-effects ordered-probit models. The authors follow Clark (1997) to some extent. The main contribution of the paper is an analysis of this gender differences among disabled workers.

**Comment 2-1:** I wonder why the authors exclude “observations with missing information for any relevant variables”. Is it to have a balanced panel dataset? The problem is they drop most of the observations. For the year 2015, for example, they keep only 19% of observations in the dataset. I think the authors should use larger sample size if possible. Having unbalanced panel data or fewer independent variables is fine to have a more representative sample. It will also increase the sample size so that the authors have larger power to reject the null hypotheses.

***Response:*** The sample size became smaller as we tried to determine the analysis sample by applying various constraints on the whole sample. First of all, the labor market participation rate of people with disabilities is considerably lower than others. In particular, since this study is limited to wage workers, about 75% of the observations were dropped out of the analysis sample. Moreover, the working age was defined as between 18 and 65, and observations whose explanatory variables related to the analysis were missing were also excluded from the analysis. This point has been described more clearly in the manuscript at page 9 (at the beginning of Section 3).

**Comment 2-2:** The authors estimate separate regressions by age, education, and occupation to explore whether expectations explain gender differences in job satisfaction whose results are presented in Table 5. I think it is difficult to draw conclusions from Table 5 because (1) whether the estimates differ is not formally tested; (2) the authors may have weak power to reject the null hypothesis in some of the specifications (the sample size is small); (3) the authors cannot draw conclusions by comparing the statistical significance (in fact, the difference between statistically significant and statistically insignificant estimates may be statistically insignificant). I think the authors should estimate regressions with interactions between gender and each of the above three variables; I think that is the more proper approach. We appreciate this comment.

***Response:*** In terms of Table 5 (in the earlier version of our paper), we divided samples to show the impact of expectations on job satisfaction. However, we believe that the points raised out by the referee were correct, and conducted re-analyses including the interaction terms as suggested, and the results were reported in Table 5. While the sum of the three different coefficients of interaction terms is large enough in absolute terms to offset the coefficient estimate of the gender variable (Model 4), the interaction between gender and education is the only one with statistical significance. Therefore, the authors revised the manuscript that the role of expectations, applied to the difference in job satisfaction between men and women in the study of non-disabled persons, cannot be fully applicable to explain the gender gap of persons with disabilities. (page 16)

**Comment 2-3:** The authors seem to only cite Clark (1997) to justify their approach of testing whether expectations explain gender differences in job satisfaction. Consider elaborating this approach further so that readers can see why this approach may help answering the research question.

***Response:*** Looking at previous studies, with several different hypotheses, researchers have attempted to explain the paradox that women show higher job satisfaction than men, even in worse working conditions. One such hypothesis concerns relative utility and expectation. In determining job satisfaction, while the absolute level of wages is important, the level of one’s wages relative to that of others can also be an important factor in determining the utility of a job. Comparison involves observing other workers and comparing them with oneself, and it is proposed that, as with relative income, relative expectations can play a role in explaining why women’s job satisfaction is higher than that of men. Clark explains that, for a variety of reasons, the expectations of older, less-educated, and non-professional women will be lower, likely leading to higher job satisfaction conditional on a set of individual and job attributes. Following suggestion by the reviewer, in this study, the discussion of expectations in the manuscript has been revised and reinforced at the last two paragraph of Section 3 (page 11 and 12). Thank you very much for this comment.

**Comment 2-4:** I think the authors should more carefully draw conclusions from their results.

In the abstract, for example, they conclude “[their] findings reveal that different work values between women and men do not account for the still significant higher job satisfaction of women, although they contribute to a partial decrease of the gender gap in job satisfaction”. I do not see evidence that different work values “contribute to a partial decrease of the gender gap” in Table 4. (The estimate “decreases” from 0.225 to 0.222 but probably they do not differ statistically.)

I am also unsure what they mean when they say job satisfaction of women is significantly higher; do they mean significantly higher statistically?

They then conclude that the hypothesis on the role of expectations is “supported by the empirical analyses that gender differentials disappear for the young, …”. But failing to reject the null hypothesis does not mean that there are no gender differences in job satisfaction; maybe they do not have enough power to reject the null hypothesis because the sample size is smaller.

Moreover, as I write above, the difference between gender differences in job satisfaction in the “young” and “older” specifications may be statistically insignificant. (I find other possible misinterpretations of their results: I do not see evidence that adding measures of job characteristics increases gender differences in job satisfaction (Table 2);

I do not see evidence that “women are more likely to put more weight on work intensity, work hours, aptitude, …” (Table 3);

job satisfaction may not necessarily decrease just because the estimate in Model 4 is smaller; see also the conclusions.)

***Response:*** Thank you for pointing out about what we missed in the earlier version of our paper. 1) First of all, women's job satisfaction is statistically significantly higher, but we must have expressed in a clearer wording. For instance, women are happier at work, *holding other explanatory variables fixed*. The body of text was appropriately modified. 2) We admit that the expression, “Women are more likely to put more weight on work intensity, work hours, aptitude,…” was confusing enough to make readers misunderstand the main point of ours. Thanks for the point, and corrected it corresponding to the data. We are also in the same line with the referee for most of the other points. 3) Particularly, without checking statistical significance, have the difference between the two groups or between models been confirmed, and appropriate corrections were made. In the revised version of our paper, we had been more cautious when interpreting the estimation results.

**Comment 2-5:** Consider using fixed-effects in addition to random-effects ordered probit model to see whether the results are robust.

***Response:*** We also considered an application of the fixed effects model, but we should not be able to include the gender variable—the key one—in the study into our model because there exists no within-group variance in sex variable.

**Comment 2-6:** Consider presenting the standard errors in tables of results, not only the asterisks.

***Response:*** We did also report the standard errors, following the suggestion.
