# Supplementary material for: Gender differences in job satisfaction among disabled workers
Source: PLoS One. 2021 Jun 4;16(6):e0252270. doi: 10.1371/journal.pone.0252270 (PMC8177467; doi:10.1371/journal.pone.0252270)
Supplement: Attachment — Submitted filename: Response To Reviewers.docx [file pone.0252270.s004.docx]

**Reviewer #1**

The revised paper has improved substantially and most of the concerns are addressed satisfactorily. This paper indeed has a potential benefit to the readers of PLOS ONE. There are only a few minor concerns which the authors might wish to address with:

The variable, 〖JS〗_it^ , need to be labelled as well (line 245). For instance, “…probability that 〖JS〗_it^ (i.e., the reported job satisfaction) is equivalent …”

The specification of cut points seems inaccurate. “…κ is a set of cut points κ_1,κ_2,…,κ_4 ; …” (line 250). This specification does not match with the Equation (3) in terms of j. According to Long (1997), the specification should be “…κ is a set of cut points κ_0,κ_2,…,κ_5 (where κ_0=-∞ & κ_5=+∞) ; …”. This will match the Equation (3).

It is stated that “…Our findings confirm that men are as happy in their workplace as women on average …” (line 324-325). From Table 3, it shows that women are happier than men. Please check.

The use of the words, “…statistically insignificantly…” (line 340) seems weird. Please rewrite this sentence.

The LR test statistics in Table 3 (Model 1) seem to incorrectly report. The extremely high values of LR test statistics (1388.089) appears to contradict with the low Wald chi-squared test statistics (2.325). Both LR test and Wald test are overall fit tests. They normally would not have huge differences in their test statistics. Please check.

Title of Table 3, 4 and 5 include the word, “standard error” in parenthesis. How about delete it and include a footnote under the tables, such as “The values in parenthesis represent standard error”?

**Response:** We addressed all of the comments raised by the reviewer above. We appreciate the referee for reviewing our paper very carefully. One thing we would like to note is that, following the suggestion, we did double-check the low Wald chi-squared test statistics by re-running our statistical package, but we are sure that it was the correct result. Lastly, in terms of the interpretation of Table 3, while Model 1 does not show gender difference, we observe the gender difference in Model 2 and 3. This is why we state “*men are as happy in their workplace as women on average because of better work conditions such as a higher salary and professional occupation, but women report* ***higher job satisfaction in identical jobs***”.

**Reviewer #3**

The previous comments by Reviewer 1 and Reviewer 2 are robustly addressed by the authors. This significantly made the results and findings more clearer and unambiguous. However, the concerns of the Reviewer 2 (Comments 2.1 and 2.4) on smaller size of the sample is an important issue which needs to be acknowledged explicitly as a research limitation in the conclusions section.

**Response:** Thank you for the comment. We added the research limitation in the conclusion section as below.

“We acknowledge some limitations in our research. We were not able to reject some of the null hypotheses, but failing to reject the null hypothesis does not mean that there are no gender differences in job satisfaction; they are unlikely to have enough power to reject the null hypothesis because of the small sample size.”
